# Preliminary evidence that light through the eyelids can suppress melatonin and phase shift dim light melatonin onset

**DOI:** 10.1186/1756-0500-5-221

**Published:** 2012-05-07

**Authors:** Mariana G Figueiro, Mark S Rea

**Affiliations:** 1 Lighting Research Center, Rensselaer Polytechnic Institute, 21 Union Street, Troy, NY, 12180, USA

**Keywords:** Dim light melatonin onset (DLMO), Light, Eyelid transmission, Circadian, Melatonin

## Abstract

**Background:**

A previous study reported a method for measuring the spectral transmittance of individual human eyelids. A prototype light mask using narrow-band “green” light (λ_max_ = 527 nm) was used to deliver light through closed eyelids in two within-subjects studies. The first study investigated whether an individual-specific light dose could suppress melatonin by 40% through the closed eyelid without disrupting sleep. The light doses were delivered at three times during the night: 1) beginning (while subjects were awake), 2) middle (during rapid eye movement (REM) sleep), and 3) end (during non-REM sleep). The second study investigated whether two individual-specific light doses expected to suppress melatonin by 30% and 60% and delivered through subjects’ closed eyelids before the time of their predicted minimum core body temperature would phase delay the timing of their dim light melatonin onset (DLMO).

**Findings:**

Compared to a dark control night, light delivered through eyelids suppressed melatonin by 36% (*p* = 0.01) after 60-minute light exposure at the beginning, 45% (*p* = 0.01) at the middle, and 56% (*p* < 0.0001) at the end of the night. In the second study, compared to a dark control night, melatonin was suppressed by 25% (*p* = 0.03) and by 45% (*p* = 0.009) and circadian phase, as measured by DLMO, was delayed by 17 minutes (*p* = 0.03) and 71 minutes (ns) after 60-minute exposures to light levels 1 and 2, respectively.

**Conclusions:**

These studies demonstrate that individual-specific doses of light delivered through closed eyelids can suppress melatonin and phase shift DLMO and may be used to treat circadian sleep disorders.

## Findings

### Introduction

Retinal light exposures at night can suppress melatonin and change circadian phase. Many studies have shown that identical light exposures delivered at different times of night will have differential impact on the circadian phase. The valence (positive or negative) of the circadian phase response curve (PRC) changes from a delaying to an advancing response to light about 1.5 hours before normal wakening, near the time of minimum core body temperature (CBT_min_). Moreover, the PRC exhibits maximal responses to light just before and just after this phase transition. Therefore, the same exact light dose can either advance or delay the circadian clock and can have a large or a small effect on circadian phase, depending upon the time of night when it is applied [1,2]. Many studies have shown these effects during the night while people are awake, but few attempts have been made to stimulate the retina with light through the closed eyelid and, in particular, while individuals are asleep.

**Figure 1:**
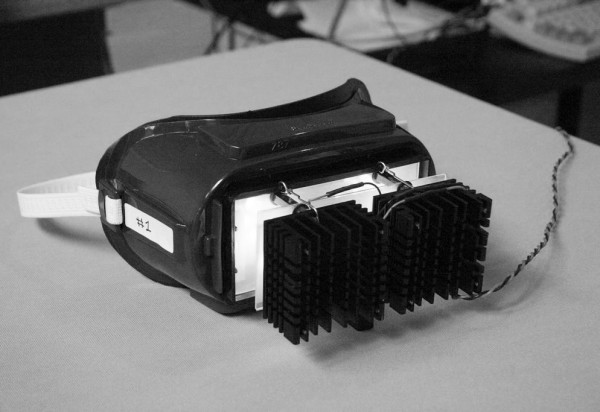
**Light mask.** Light mask used in the two studies to deliver individualized dose of 527 nm, “green” light.

Recently, Bierman et al. [3] measured and modeled eyelid spectral transmittance, showing high attenuation to short-wavelength light where the human circadian system is most sensitive. On average, the closed eyelid attenuates circadian-effective light by approximately two orders of magnitude, but individual spectral transmittances can vary by one order of magnitude. Thus, to precisely dose light for circadian stimulation through a person’s eyelids, it is necessary to prescribe the spectral irradiance on the eyelid as it will be attenuated by the spectral transmittance of that person’s eyelids. A special light mask (Figure 1) was developed for the present two studies to provide individualized doses of light for the stimulation of the circadian system through closed eyelids. The goal of the first study was to determine if the light delivered through a person’s eyelids could predictably suppress nocturnal melatonin while the person slept. The goal of the second study was to reinforce the nocturnal melatonin suppression results of the first study and to extend those findings to determine if an individualized light dose could change circadian phase. By combining the phototransduction model by Rea et al. [4] with individually-measured spectral transmittance functions, it was possible to develop an individually-prescribed dose of light for stimulating the retina through the closed eyelids. The spectral power distribution of the light exposure was optimized for circadian system stimulation [4], transmission through the eyelid [3], and minimization of blue-light hazard [5], taking into consideration the practical performance characteristics of commercially available light-emitting diodes (LEDs). Polysomnography (PSG) was used to time the delivery of the light dose and to measure its effect on sleep efficiency.

### Methods

#### Experiment 1: Acute melatonin suppression during sleep

##### Subjects

Six subjects (three females) from 40–65 years of age (mean ± standard deviation = 48.6 ± 9.33 years) agreed to participate in the study and were free from any major health problems, such as cardiovascular disease, diabetes, or high blood pressure. Subjects were excluded from the experiment if they were taking over-the-counter melatonin, antidepressants, sleep medicine, beta blockers, or hormone replacement therapy drugs. Each subject participated in a two-night protocol separated by one week. Subjects were asked to keep a regular schedule (bed times before 23:00 h and wake-up times no later than 08:00 h) during the weeks prior to the night of data collection. The night prior to the study, subjects were asked to go to bed by 22:00 h. To verify compliance, each subject wore a wrist actigraph (Actiwatch Spectrum IPX7, Philips Respironics, Pittsburgh, PA, USA) and kept a sleep log starting one week prior to the experiment. Subjects were asked to avoid naps and to refrain from consuming caffeine on the day of the experiment. All subjects completed a consent form approved by the Rensselaer Polytechnic Institute Institutional Review Board [6] and were paid for their participation. The research was conducted according to the principles expressed in the Declaration of Helsinki [7].

##### Light mask

Figure 1 shows the light mask used in the present studies. Green LEDs (λ_max_ = 527 nm, full-width-half-maximum = 36 nm) were selected as the light source because this wavelength optimizes eyelid spectral transmission [3] with the spectral sensitivity of the human circadian system [4]. Also as an important consideration in light source selection, exposure to high radiances of this wavelength limits blue light hazard risk relative to shorter wavelengths [5]. Machined aluminum heat-sinks were used with the prototype mask to dissipate the heat generated by the light sources. The light generated by the prototype mask was controlled by a program that increased light levels gradually over two minutes from zero to the prescribed light level, with the assumption that this temporal ramp would avoid “startling” the subjects with the light dose while they were asleep.

##### Lighting conditions

Individualized light doses were determined from the measured spectral transmittance of one eyelid of each subject and the calculated amount of the green light irradiating closed eyelids needed to suppress melatonin by 40% according to the model by Rea et al. [4]. For the calculations of circadian stimulus needed for 40% suppression, a standard observer was assumed with the following characteristics: pupil diameter = 2.3 mm, circadian time = 2 hours before predicted CBT_min_ (approximately 03:00 h), exposure duration = 60 minutes. Effective light doses (irradiance x time) on the closed eyelids of each subject were calculated using the individual-specific eyelid spectral transmittance data obtained on night 1 utilizing the methods described by Bierman et al. [3]. Briefly, a nurse gently lifted each subject’s eyelid from the cornea and then placed it between the light source and light sensor of a special “wand” that quickly measures the eyelid spectral transmittance. As described by Bierman et al., a measurement takes less than 5 s, with no discomfort whatsoever to the subject. The light mask was then calibrated and its output adjusted for each subject. The nurse had some difficulty collecting eyelid measurements from one subject due to excessive squinting and wrinkling of the eyelid during measurement. This reaction was mostly due to fear that the apparatus was going to hurt him. For this subject, the individually-prescribed dose of light was based upon the median eyelid transmission of eighteen subjects previously measured by Bierman and colleagues [3]. The irradiances predicted to be required by each subject to obtain 40% melatonin suppression varied from 33 to 85 W/m^2^ (17,000 to 50,000 lux) at the closed eyelid.

##### Procedure

Each subject participated in a two-night protocol during the months of October to December 2009. On the days of the experiment, subjects were asked to arrive at the laboratory at 22:00 h. Upon arrival to the laboratory, illuminated by conventional ceiling-mounted fluorescent light sources (OCTRON XP, 3500 K lamp, Sylvania, Danvers, MA, USA) delivering approximately 100 lux at the cornea, subjects were asked to wear dark glasses (transmission < 10%) while being prepared for the experiment. Subjects were fitted with electrodes for the recording of electroencephalography, electrooculography, and electromyography used in PSG (Alice System, Philips Respironics, Pittsburgh, PA, USA). Using the International 10/20 system, scalp electrodes were placed at C3, C4, O1, O2, A1, and A2, with a ground electrode in the middle of the forehead. For electromyography, one electrode was placed centrally on the chin and the other on the submentalis muscle. Standard placement was used for right and left electrooculography. A registered nurse inserted an indwelling catheter into one of the subjects’ veins. The room lights were turned off at 23:00 h and dim red light (λ_max_ = 640 nm), < 1 lux at the cornea, was provided to the room by LEDs for the remainder of the night. Subjects were then asked to sit in a recliner chair and were outfitted with the light mask.

On both nights, blood samples for melatonin assays were collected during three sessions: 1) the initial part of the night while subjects were awake, 2) the middle of the night while subjects were asleep in non-rapid eye movement (non-REM), and 3) the latter part of the night while subjects were asleep during REM. The exact collection times while subjects were asleep were dependent upon their sleep stages, which were continuously monitored throughout the entire experiment by the experimenters using PSG.

During the first session, subjects were awake, wearing the light mask. On the first night, the light mask was not energized, and data from this night served as a baseline. On the second night, subjects were again awake and wore the light mask during the first session, but this time light was delivered through the closed eyelids from 23:30 h until 00:30 h. Blood samples were drawn from an indwelling catheter every 20 minutes starting 20 minutes prior to putting on the light mask. Two samples were taken before the light mask was energized and four samples were taken during the light exposure.

At 01:15 h, subjects began the second and third sessions, involving data collection while they were asleep in a reclining chair, wearing the light mask. During the second session, once a subject entered stage 2 sleep, blood samples were taken every 30 minutes for a total of three samples. On night 1, the three samples were all taken while wearing the un-energized light mask, whereas on night 2, one blood sample was obtained before energizing the light mask and two samples were obtained 30 minutes and 60 minutes after light onset. Session three on both nights began at the onset of REM sleep. On night 1, three blood samples were drawn when the light mask was not energized, whereas on night 2, one sample was obtained before the light mask was to be energized for 60 minutes. Subsequent samples were obtained 30 minutes and 60 minutes later; on night 2, the samples were taken during light exposure. The applications of the light stimuli during the second and third sessions of both nights were separated by at least 45 minutes to ensure that melatonin concentrations would rise after the first light exposure.

##### Data analyses

Blood samples were spun for 15 minutes at 1000x g for plasma extraction on the night of sample collection. Plasma samples were then frozen (−20°C) and later sent to a laboratory (Pharmasan, Osceola, WI, USA) for melatonin radioimmunoassay. The sensitivity of the plasma assay was 3.5 pg/ml and the intra- and inter-assay coefficients of variability were 8.1% and 14.8%, respectively.

A 3 (sleep condition: awake, non-REM, and REM) x 2 (light mask dark/control and light exposure) analysis of variance (ANOVA) was used to compare melatonin concentrations after 60 minutes of light exposure or of darkness. Two-tailed, paired Student’s t tests were used to further compare the significant main effects and interactions.

Melatonin suppression was calculated using the ratio of the melatonin concentrations obtained on night 1 (dark) and those obtained near the same clock time on night 2 (light on). Melatonin concentrations were normalized to minimize unwanted differences among individuals. The normalization factors for every subject were determined by dividing the mean of melatonin concentration for all subjects and all experimental conditions by the mean of each subject’s melatonin concentrations for all experimental conditions. All the data from a subject were then multiplied by their own normalization factor. Two-tailed, One-Sample T tests were used to determine whether melatonin suppressions after light exposures were significantly different than zero. A criterion level for Type I errors at a probability of 0.05 was used for all of the statistical tests.

#### Experiment 2: Acute melatonin suppression and circadian phase shifts

##### Subjects

Seven subjects (two females) with mean ± standard deviation age 23 ± 7 years agreed to participate in the study, were screened for major health problems, and reported abstention from pharmaceuticals or medications. All subjects provided written informed consent approved by Rensselaer’s Institutional Review Board [6] and were paid for their participation. The research was conducted according to the principles expressed in the Declaration of Helsinki [7]. Subjects were asked to refrain from alcohol and caffeine on the days of the experiment.

Every potential subject completed a Munich Chronotype Questionnaire prior to the study, and those who were late or extremely late chronotypes were excluded from the experiment [8]. All of the selected subjects were asked to maintain a regular sleep/wake schedule starting one week prior to the study and continuing during the three weeks of the experiment to minimize differences in circadian phase among subjects. To help ensure compliance, participants completed a sleep/wake diary during all weeks of the experiment, and wore wrist actigraphs (Actiwatch Spectrum IPX7, Philips Respironics, Pittsburgh, PA, USA) on the non-dominant wrist starting one week prior to the first night in the laboratory. These data were examined before subjects were approved for participation in data collection.

##### Procedures

Experiment 2 was conducted from June to August 2010. All subjects arrived at the laboratory between 18:30 h and 20:30 h on a Thursday, and each session concluded approximately 30 hours later, between 00:00 h and 02:00 h on a Saturday. Subjects’ arrival times were based on their estimated dim light melatonin onset (DLMO), which was calculated using their self-reported sleep times [9]. The protocol consisted of three experimental conditions, each presented during a separate session, at least one week apart from each other. The light masks used were identical to those in Experiment 1. The first night was a control (dark) night when eyelid spectral transmittances were measured for each subject. Subjects wore the un-energized light mask while they slept. The subjects were presented the two other experimental conditions over the course of two weeks, in a counterbalanced order. Light level 1 (LL1) was a lower light level and light level 2 (LL2) was a higher light level. Based on calculations performed using the model of human circadian phototransduction by Rea and colleagues [4] and the individually-measured eyelid spectral transmittances, LL2 delivered from the light mask was set for every subject to suppress nocturnal melatonin by 60% and LL1 was set to suppress nocturnal melatonin by 30%.

The room lights were turned off at 18:30 h and dim red (λ_max_ = 640 nm) light, <1 lux at the cornea, was provided to the room by LEDs for the remainder of the night. Starting from 18:50 h to 20:50 h depending on the individual estimated DLMO, saliva samples were collected every 20 minutes for 5 hours, to assess DLMO phase. Saliva collection was done using the Salivette system (Sarstedt, Newton, NC, USA). To prevent contamination of the saliva samples, the subjects were only allowed to sip water right after each saliva collection time. No drinking was allowed 15 minutes prior to each saliva sample collection. No eating was allowed during DLMO data collection period.

Before being allowed to sleep, a registered nurse inserted an indwelling catheter into one of the subjects’ veins. After DLMO collection ended, the subjects were given the opportunity to sleep and were awakened the following morning at 09:00 h. While the subjects were sleeping, the light mask was activated two hours prior to their estimated CBT_min_, which was defined for this experiment as equivalent to seven hours after predicted DLMO. This should have placed the light exposure in the delay portion of the PRC. Therefore, DLMO was expected to be delayed on the second night of the protocol with respect to that measured on the first (dark, control) night. The light always remained on for 60 minutes. Two blood samples were drawn from the indwelling catheter 40 minutes and 20 minutes prior to light exposure and 30 minutes and 60 minutes after light onset. Thus, a total of four blood samples were collected while subjects slept. After the fourth blood sample was collected, the indwelling catheter and light mask were removed. If subjects were awake, they were allowed to return to sleep.

From 09:00 h until 18:30 h, subjects remained in dim red light and were allowed to perform normal computer work, read, and play games. Electronic devices (i.e., computers, phones, portable media players) were dimmed to the lowest possible brightness and were covered with an orange filter (Roscolux #21 Golden Amber, Rosco, Stamford, CT, USA) that removed short wavelengths. Transmittance through the filters from 380 to 550 nm was 2.1%. Napping was not allowed during the experiment. Breakfast, lunch, and dinner were offered at specific times. Subjects were asked to brush their teeth after dinner, which was offered 60 minutes prior to data collection.

Saliva samples were collected every 20 minutes for 6 hours, starting at 19:00 h or at 20:50 h to assess DLMO phase. After the last saliva sample was collected, subjects were allowed to go home.

##### Data analyses and phase shift calculations

Saliva samples were frozen (−20°C) after collection and later sent to a laboratory (Pharmasan, Osceola, WI, USA) for melatonin radioimmunoassay. The sensitivity of the saliva assay was 0.7 pg/ml and the intra- and inter-assay coefficients of variability were 12.1% and 13.2%, respectively.

##### Melatonin concentrations and acute melatonin suppression

A 3 (dark, LL1, and LL2) x 4 (sample collection times) ANOVA was performed on the normalized melatonin concentrations. Two-tailed, paired Student’s t tests were used to further compare the significant main effects and interactions.

Melatonin suppression was calculated using the ratio of the melatonin concentrations obtained on night 1 (dark) and those obtained near the same clock time on night 2 (energized light mask). Two-tailed, One-Sample T tests were used to determine whether melatonin suppressions after light exposures were significantly different than zero. For all of the statistical tests, a criterion probability level of 0.05 for a Type I error was used.

##### Phase shifting

Melatonin profiles for each subject on each night (dark, LL1, and LL2) were fitted with a locally weighted least-squares curve [10,11]. DLMO threshold was calculated by taking the mean of the five lowest continuous melatonin concentrations plus 15% of the five highest continuous values. DLMO for each melatonin profile was the time (as determined by linear interpolation) that the fitted curve reached and remained above the calculated DLMO threshold [12]. If the fitted curve had not reached and remained above the calculated DLMO threshold by the end of the data collection period, DLMO time was taken be at the last data collection time. Phase shifting was determined by subtracting the time of DLMO determined for the control night (Saturday night that subjects remained in the dark) from the time of DLMO determined for the light exposure night (Saturday night following nocturnal light exposure). As an arbitrary convention, a negative remainder indicates that the nocturnal light exposure was associated with a delay in circadian phase.

Two-tailed, One-Sample T tests were used to determine whether the phase shifts obtained after exposure to LL1 and LL2 were significantly different than zero (i.e., no different than a natural phase shift in the dark).

### Results

#### Experiment 1: Acute melatonin suppression during sleep

**Figure 2:**
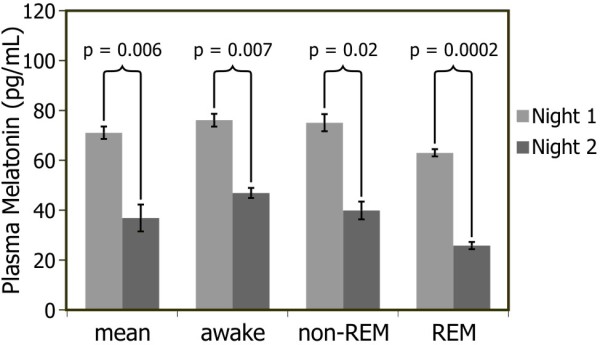
**Normalized melatonin concentrations for Experiment 1.** Mean ± SEM melatonin concentrations for all subjects in the first study. As shown on the far left, the mean melatonin concentrations on night 2 (after light exposure) were significantly lower than on night 1 (dark night); mean suppression after 60-minute light exposure was 46%. The melatonin concentrations on night 2 were significantly lower than the comparable concentrations on night 1 while subjects were awake (mean suppression after 60-minute light exposure was 36%), during non-REM sleep (mean suppression 45%), and REM sleep (mean suppression after 60-minute light exposure was 56%).

Figure 2 shows the overall mean ± standard error of the mean (SEM) normalized melatonin concentrations (pg/ml) for nights 1 (un-energized light mask) and 2 (energized light mask) as well as the melatonin concentrations at the three different times of the night and sleep stages (start/awake, middle/non-REM, and end/REM). A 3 (time of night/sleep stage) x 2 (light mask energization) ANOVA revealed a significant main effect of light mask energization (F_1,5_ = 21.2; *p* = 0.006). The mean ± SEM normalized melatonin concentration on night 1 was 71 ± 16 pg/ml, while on night 2 it was 36 ± 16 pg/ml. The main effect of time of night/sleep stage was not statistically significant (F_2,10_ = 3.6; *p* = 0.07), nor was the interaction between the two variables (F_2,10_ = 0.3; *p* = 0.8). Simple two-tailed, paired Student’s t tests were performed comparing melatonin concentrations at each of the three times of the night/sleep stages (start/awake, middle/non-REM, and end/REM), with and without light mask energization. There was a significant difference in melatonin concentrations between nights 1 and 2 when subjects were awake (*p* = 0.007), during non-REM (*p* = 0.02), and during REM (*p* = 0.0002) sleep.

The mean ± SEM suppression after 60-minute light exposure was 36 ± 9% in the first part of the night (awake), 45 ± 12% in the middle part of the night (non-REM), 56 ± 7% in the latter part of the night (REM). Melatonin suppression by the light through the eyelids was very close to predicted values (40%) during the middle of the night. Two-tailed, One-Sample T tests revealed that melatonin suppression was significantly greater than zero after light exposure at the start (*p* = 0.01), middle (*p* = 0.01), and end of the night (*p*< 0.0001).

##### PSG Results

Based upon the PSG reports, the mean sleep efficiency on night 1 was 76 ± 5%, while on night 2 (energized light mask) was 78 ± 5%. The mean total sleep time was 200 ± 15 min on night 1 and 222 ± 15 min on night 2, and the mean wakefulness after sleep onset was 83 ± 22 min on night 1 and 74 ± 21 min on night 2. During the 1-hr light period in the third session of night 2 (REM sleep), subjects were awake for only 6 ± 3 min.

#### Experiment 2: Acute melatonin suppression and circadian phase shifts

##### Melatonin concentrations

**Figure 3:**
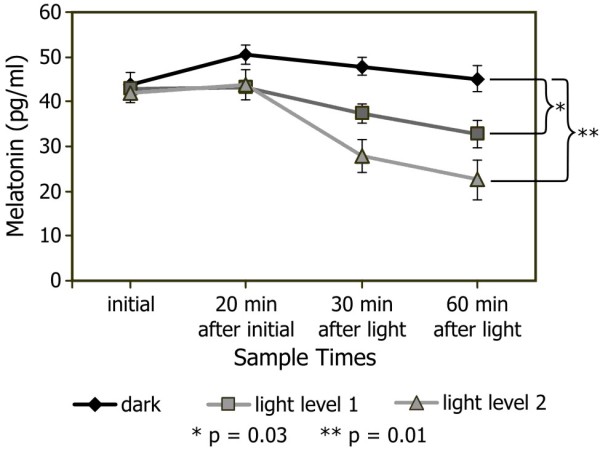
**Normalized melatonin concentrations for Experiment 2.** Mean ± SEM melatonin concentrations for the seven subjects who completed the second, three-night, within-subjects study. The first two measurements were collected prior to turning on the mask. The last two measurements were collected 30 and 60 minutes after the light mask was energized (except for the dark, control night when the mask remained off for the duration of the experiment). Nocturnal melatonin suppression levels were 25% and 45% after 60-minute light exposures to the LL1 and LL2, respectively.

Figure 3 shows the mean normalized melatonin concentrations. The ANOVA revealed the main effect of light was not statistically significant (F_2,10_ = 3.5; *p* = 0.07), but there was a significant main effect of sample time (F_3,15_ = 20.1, *p* and there was a significant interaction between 0.0001) and there was a significant interaction between the two variables (F_6,30_ = 4.8; *p* = 0.002).

As shown in Figure 3, LL2 was a stronger stimulus for suppressing melatonin than LL1. To further analyze the significant light x sample collection time interaction, two-tailed, paired Student’s t tests revealed that melatonin concentrations after 60 minutes in the dark (control night) were significantly higher than melatonin concentrations after 60 minutes of exposure to LL1 (*p* = 0.03) and to LL2 (*p* = 0.01).

Nocturnal melatonin suppression was calculated by taking the ratio of the melatonin concentrations after exposure to LL1 or LL2 and melatonin concentrations in the dark/control night after 60-minute exposures. As expected, suppression was greater after exposure to LL2 than after exposure to LL1; the mean suppression levels were 25 ± 9% for LL1 and 45 ± 12% for LL2. Two-tailed, One-Sample T tests revealed that melatonin suppressions after exposure to LL1 and LL2 were significantly different than zero (*p* = 0.03 for LL1 and *p* = 0.009 for LL2).

##### Phase shifting

**Figure 4:**
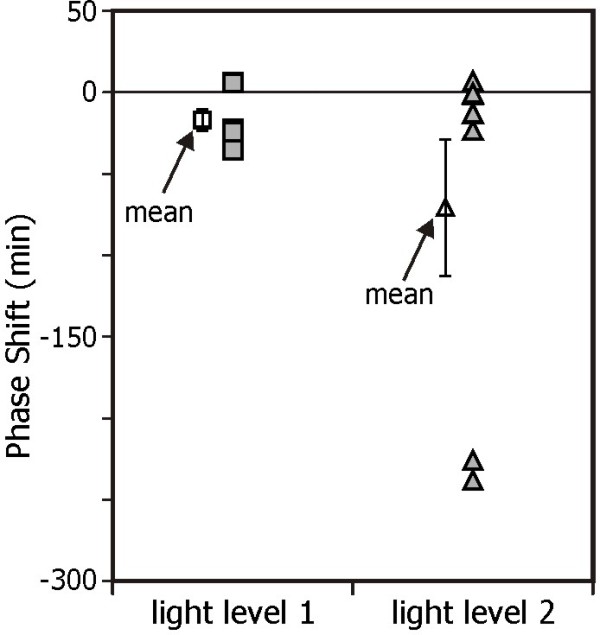
**Change in circadian phase.** Change in circadian phase, as measured by DLMO, for seven subjects who completed the second, three-night study. A negative value indicates that DLMO was delayed relative to the dark, control night as a result of the light exposure. Mean ± SEM for the low and high light levels are also shown. Note that some subjects had similar phase shifts and their data are overlapping in the graph.

Figure 4 shows the estimated change in DLMO, in minutes, induced by the light exposures relative to the dark control night. Subjects 23 and 25 did not have melatonin concentrations above the DLMO threshold by 02:10 h, so, as noted above, the time of the last saliva sample collection was used as a conservative estimate of their DLMO times. The mean ± SEM relative phase shift was −17 ± 6 minutes after exposure to LL1 and −70 ± 41 minutes after exposure to LL2. Two-tailed, One-Sample T tests revealed that LL1 was significantly different than zero (p = 0.032) but LL2 was not (*p* = 0.14), despite having a greater mean phase shift. The median phase shift was −24 minutes after exposure to LL1 and −13 minutes after exposure to LL2.

### Discussion

The results of both studies showed that individually-prescribed light stimuli delivered through closed eyelids were sufficient to suppress melatonin while subjects were awake and while they were asleep. Importantly, the prescribed light levels, estimated using the eyelid spectral transmission values at 527 nm for each subject and the model of human circadian phototransduction by Rea and colleagues [4], resulted in average melatonin suppression levels close to the predicted values. It should be noted, however, that the prescribed light delivered by the light mask did not suppress melatonin for one of the subjects in the first study. According to her eyelid measurements, she had high eyelid transmittance, so she had the lowest prescribed light dose in either study. She also had unusually low melatonin concentrations in the dark. It may be that the low prescribed light level and/or her low melatonin concentrations contributed to her negative findings. She also described herself as a “light sleeper” which may have also affected her results. Her PSG report showed numerous awakening episodes throughout the course of the night, with a total of 100 micro arousals compared to a mean of 42 micro arousals per night experienced by the other subjects. Notwithstanding the data for this one subject, the eyelid spectral transmission measurement procedure together with the model of nocturnal melatonin suppression can be used with some confidence to prescribe an individual light dose through a person’s closed eyelid.

The present melatonin suppression findings are not necessarily inconsistent with others in the literature where light was delivered to the retina through closed eyelids. Hatonen et al. [13] exposed subjects to 2,000 lux of white light (True-Lite fluorescent lamps, 5500 K) at the eyelid, with eyes opened and closed, and were able to demonstrate significant melatonin suppression only in the setting of opened eyes. Two out of eight subjects decreased their melatonin concentrations by the light exposure through the closed eyelids, but on average, melatonin concentrations were not significantly different from the control night (dim light). These results are consistent with our predictions based upon the average eyelid transmission estimates from Bierman et al. [3]. Using these average eyelid transmission values, subjects should have received approximately 40 lux at the cornea from the 5500 K light source, mostly from long wavelengths, so the eyelid-filtered “white” light would be largely ineffective for suppressing melatonin and activating the circadian system. Based upon the high variability found in eyelid transmission among individuals [3], it is not unexpected that two of the eight subjects in the Hatonen et al. study [13] could have suppressed melatonin. If these two subjects indeed had high eyelid transmittances, the white light might have provided a sufficient light stimulus to suppress some melatonin through their closed eyelids.

Phase shifting responses to the light delivered by the light mask were shown in the second study. Unlike the close correspondence between the predicted and the observed melatonin suppression, the observed magnitudes and directions of the phase shifts in DLMO were more likely to differ from the predicted magnitudes and directions. As is commonly accepted [9,14], personal sleep log data were used to prescribe the individualized times of light dose delivery to achieve the desired changes in circadian phases. As shown by Crowley et al. [14] and Burgess and Eastman [9], however, actual DLMO cannot be precisely predicted from sleep logs, particularly for subjects on a strict schedule, as in this study. If, for example, actual DLMO is advanced a couple hours with respect to predicted DLMO, a light dose prescribed for a modest phase delay would be applied closer to the cross-point in the PRC, resulting in a much larger phase delay than expected, or even a phase advance. Thus, the observed magnitudes and directions of phase shift will be increasingly more variable the closer the light is applied near the cross-point in the PRC.

As pointed out in the results section, two subjects in this experiment had much stronger phase delay responses to LL2 than the other subjects. The only night we were not able to determine DLMO for these two subjects was on night 2 after exposure to LL2 (post treatment night). DLMO was determined for these two subjects in all other nights, including the dark, control night. Consistent with the above rationale, the observed DLMO for both of these subjects on the dark, control night was much earlier than the DLMO predicted from the sleep logs. One of these subjects who showed a very large phase delay to LL2 showed a phase advance to LL1. Thus, light stimuli were likely delivered to these two subjects at times closer to the respective cross-points of their PRC. A strong circadian stimulus, such as the one delivered by LL2, if given on the phase delay side, but close to the cross-point, may lead to a very large phase delay, similar to the ones observed after exposure to LL2 in these two subjects. If the same stimulus is, however, split between the two sides of the cross-point (i.e., advance and delay portions of the PRC), it can result in a smaller net phase shifting because it may have induced some phase delay and some phase advance, reducing the overall effect of the light exposure. Yet, a third subject who had a much earlier observed DLMO than was predicted from the sleep logs exhibited phase advances following exposures to both LL1 and LL2, as would be expected if the majority of the light exposure was delivered after the cross-point of the PRC. This subject exhibited a small phase delay following the control (dark) night, which would be consistent with a free running period slightly greater than 24 hours. His light exposure ended close to 05:15 h, so it is quite possible that at least some of the light exposure was in the phase advance portion of his PRC, which would explain his net phase advance in DLMO.

Although it is very appealing to consider using light delivery through the eyelid while people sleep to maximize its effectiveness for phase shifting, until we are better able to predict the timing of the cross-point of the PRC, light delivery with the mask should be early in the night for a phase delay response or about an hour before wake-up times for phase advance to assure that light is delivered in the correct side of the PRC. Again, these results clearly show the ability of light delivered through the eyelid to suppress melatonin and phase shift DLMO; however, the ability to precisely control magnitude and direction is compromised when using sleep log data.

One previous study also looked at circadian phase shifting by light through closed eyelids. Cole and colleagues [15] used a light mask that exposed subjects to 2,700 lux of white light on closed eyelids. In comparison to a placebo light (0.1 lux of red light), they demonstrated significant melatonin phase shifts only among select patients with delayed sleep phase disorder, but not among subjects who are normally entrained to the 24-hr light/dark cycle. The authors suggest that the light mask in that study was energized 4 hours prior to the scheduled time of rising, so the long duration may have illuminated a less responsive portion of the PRC or it may have induced some phase delay prior to inducing phase advances, reducing the overall effect of the light treatment. Based upon the average eyelid transmission values from Bierman and colleagues [3], however, the white light would have delivered approximately 57 lux at the cornea. Similar to the post hoc inferences drawn from Hatonen et al. [13] study of melatonin suppression, the marginal phase-shifting effects found by Cole and colleagues may be have been due, at least in part, to the low, eyelid-filtered light levels on the cornea and the high individual differences in eyelid transmittances.

Finally, it is important to emphasize that most subjects were able to sleep while light was being applied through their eyelids. Observations by the experimenters were consistent with participants’ subjective impressions, as well as with the PSG scores in the first study. Although some subjects briefly woke up when the mask reached full light output the first time at night, they went back to sleep shortly thereafter. The light did not awaken most of the subjects during REM sleep. In fact, at the conclusion of the experiment, when asked how many times the light mask was turned on at night, all subjects said they only noticed it once. In some instances subjects were awakened when the nurse was drawing the blood, but again, they reported going back to sleep right away. In both studies, none of the subjects reported opening their eyes during the light exposure, especially because they were instructed to avoid doing so because the light would be uncomfortably bright. Furthermore, based on observations by the experimenters from both studies, all subjects were asleep (i.e., snoring or not exhibiting activity) a few minutes after the blood samples were collected and when masks were energized. Even if they had seen the light flash directly, the radiance would be uncomfortably bright and natural photophobic response would cause them to close their eyes immediately. The fact that we used a 527-nm light minimized the risk for blue light hazard if subjects briefly opened their eyes. Therefore, even if subjects had briefly opened their eyes during the light exposure period, this brief light exposure was determined to be below the threshold for blue light hazard. In fact, the Institutional Review Board reviewed and approved our calculations performed for eyes open and eyes closed. If subjects opened their eyes during the light exposure, the brief, but very bright flash of light may have, however, resulted in some phase shifting or acute melatonin suppression. We do not believe this was the case given that, as mentioned before, none of the subjects reported opening their eyes and, if awakened, all subjects were instructed to keep their eyes closed at all times. Moreover, it should be noted that a single, very brief light exposure would not be expected to have much effect on phase shifting. Zeitzer et al. [16] showed phase shifting after 60 exposures to brief, very bright light pulses, but not after a single exposure. Thus, it is unlikely that the results observed here are artifacts of brief eye openings during the study.

In summary, although the results are very clear with respect to suppressing melatonin through closed eyelids, our ability to predict the magnitude and direction of phase shifting was much more variable, probably because circadian phase cannot be precisely predicted from sleep log data. The closer light is delivered to the time when the PRC changes from phase delay to phase advance, the more variable the magnitude and the direction of the phase shift will be. Nevertheless, a more in-depth set of studies needs to be conducted examining potential artifacts of light exposure through closed eyelids and examining other populations, such as patients with actual circadian sleep disorders. A light mask similar to the one tested here can possibly be used to effectively treat circadian sleep disorders, such as delayed sleep phase disorder experienced by young adults or early morning chronic insomnia experienced by older adults. Moreover, user acceptance of a light treatment device for field use also has to be conducted. Based on our observations in the laboratory and on informal interviews the following day, subjects seemed to be able to sleep through the light exposure period without waking or experiencing discomfort. Considering that the light mask employed in the present study was not particularly comfortable, these results are quite encouraging for further experimentation and product development. A light mask that delivers stimuli through closed eyelids (during sleep) may offer a practical means of circumventing barriers to phototherapy compliance and can lead to the implementation of an economical, simple, non-pharmacological treatment modality for circadian sleep disorders. Future research should further test the light mask in clinical applications.

## Abbreviations

PRC = Phase response curve; CBTmin = Minimum core body temperature; LED = Light-emitting diode; PSG = Polysomnography; SEM = Standard error of the mean; REM = Rapid eye movement; ANOVA = Analysis of variance; LL1 and LL2 = Light level 1 and Light level 2; DLMO = Dim light melatonin onset.

## Competing interests

The authors have no competing interests to declare.

## Authors’ contributions

MGF participated in the design of the experiment, data collection, data analyses, and drafting the manuscript. MSR participated in the design of the experiment, data analyses, and manuscript writing. Both authors read and approved the final manuscript.
